# How to Make Your Fish Work for You: Tips from Ethology and Ecology for Finding Appropriate Unconditioned Stimuli for Learning Studies with *Zebrafish*

**DOI:** 10.3390/ani16050736

**Published:** 2026-02-27

**Authors:** Robert Gerlai

**Affiliations:** Department of Psychological & Brain Sciences, University of Toronto Mississauga, Mississauga, ON L5L 1C6, Canada; robert_gerlai@yahoo.com or robert.gerlai@utoronto.ca

**Keywords:** associative learning and memory, classical conditioning, ethology, motivation, reward, *zebrafish*

## Abstract

A key requirement of associative learning studies is the ability to motivate the subject to acquire memory of the conditioned stimulus–unconditioned stimulus (CS–US) association. Although *zebrafish* have been found capable of acquiring CS–US associative memory, in many studies, the fish failed to learn. One reason for the failure, I argue in this perspective article, is that we do not yet know how to motivate *zebrafish*. I illustrate this problem using examples, and offer some solutions, based upon results obtained in my own laboratory for appetitive associative learning tasks for *zebrafish*. I highlight the value of considering the ethology and ecology of the *zebrafish*. I discuss why food may have been an ineffective US for *zebrafish*. I provide examples for how to improve the rewarding properties of food based upon the foraging behaviour of *zebrafish* in nature. I discuss the efforts to identify alternative USs, including the sight of conspecifics or the presence of other ecologically relevant stimuli. I theorize about conflicting motivators in *zebrafish* learning studies, including the effect of human handling versus that of experimenter-controlled USs. I conclude that systematic analyses of different USs are needed, along with detailed studies on how they may be optimized for the analysis of learning and memory in *zebrafish*.

## Simple Summary

The *zebrafish* is gaining popularity in a variety of subfields of biology. One of these rapidly evolving fields is behavioural neuroscience. In particular, for the analysis of learning and memory, the *zebrafish* has been proposed as an alternative to traditional rodent models. Although *zebrafish* have been demonstrated to be able to learn and remember, perhaps due to the publication bias against non-significant results, failed learning and memory studies, including our own, remained unreported. In this perspective article, I argue that an important, and unresolved, problem may have contributed to these failures: our lack of understanding of how to properly motivate *zebrafish*. I illustrate the issues using studies conducted in my own laboratory. I discuss paradoxical findings, e.g., why making *zebrafish* hungry reduces their motivation to learn to find food. I also elaborate on how borrowing from nature could help us find better ways to motivate *zebrafish* and thus design more appropriate learning and memory tasks. I also discuss the deleterious effects of confounding factors, including those of human handling, and briefly mention potential ways to minimize such effects. Despite the above complexities, I present an optimistic view for the future of *zebrafish* in both basic and translational research.

## 1. Introduction

Learning and memory are fundamentally important features of our own species and those of numerous non-human animal species as well. These cognitive phenomena allow human and non-human animals to quickly adapt to changing environmental conditions, i.e., to adjust their behaviour based upon past experience. Learning and memory have been extensively studied from several standpoints of biology; i.e., their psychological, neurobiological, and molecular mechanisms have been thoroughly investigated. Discovering such mechanisms is not only important from a basic science perspective but also crucial for the human clinic, as a large number of patients suffer from a variety of brain disorders associated with the impairment of these cognitive processes (e.g., [1,2,3]). However, despite decades of research into the mechanisms of learning and memory, and despite concerted efforts to develop treatments for brain disorders associated with learning and memory impairment, a lot remains to be discovered. For example, although we know hundreds of molecular players (genes and the proteins they encode) involved in learning and memory, it is speculated that the number of such players is an order of magnitude larger than what we have discovered so far (compare [4] and [5]; also, see [6,7] for further discussion). Similarly, in the clinical domain, we still do not have effective treatment options for neurodegenerative diseases that rob millions of patients of their memories (e.g., [8,9,10]), again, likely due to lack of our understanding of molecular and other neurobiological mechanisms of memory. To ameliorate these problems, i.e., to speed up discovery, numerous animal models have been proposed, among which the *zebrafish* is one of the newer ones ([6,7,11,12,13,14], and references therein).

The *zebrafish* is still considered a relative newcomer in behavioural neuroscience, including in research on learning and memory, as one can find orders of magnitude more studies on these topics with mice and rats. Nevertheless, this species is becoming an increasingly popular subject in the latter fields [6,7,11,12,13,14]. This popularity is due to several factors. The first one is practicality. This teleost is small and is easy to keep and breed in large numbers in a cost-effective manner in the laboratory. Another reason is that it represents a reductionist approach. It is much simpler than humans, or the classical biomedical research organisms, rodents. Although mammalian model organisms are much more closely related to humans, fish, including the *zebrafish*, possess numerous evolutionarily conserved features that make these species translationally relevant for biomedical research. These similarities range, for example, from the nucleotide sequences of fish and human genes, to neurotransmitter systems and the connectome of the brains of these species. This does not come as a surprise to evolutionary biologists. The last common ancestor between mammals and fish lived about 0.4 billion years ago; i.e., fish and mammals shared their biological evolution for the first 3.4 billion years since life arose on Earth about 3.8 billion years ago, a topic I have discussed elsewhere [7,15]. Thus, both from a basic science and translational perspectives, the *zebrafish* appears to be a reasonably good compromise between practical simplicity and biological system complexity [6,7,16].

In order for one to create a good animal model for a brain disorder of learning and memory, or for one to study the mechanisms of these phenomena, one has to first establish appropriate behavioural paradigms [13,17]. Numerous learning and memory paradigms have been developed for the *zebrafish* (for recent reviews, see [18,19]). Using these paradigms, we, and others as well, have found *zebrafish* to be able to acquire and recall the association between a conditioned stimulus (CS) and an unconditioned stimulus (CS) [20,21,22,23,24,25,26,27,28]. Briefly, by now, several studies have demonstrated the good associative learning and memory capabilities of *zebrafish* (recently reviewed in [18,19]). However, hidden behind these successes is a rarely discussed issue: in numerous learning studies, *zebrafish* did not perform well. Perhaps because of the publication bias towards positive (statistically significant) results, the actual number of such failures is hard to estimate [29,30]. However, based upon my experience with studies conducted in my laboratory, the problems with *zebrafish* in learning studies are substantial. In this perspective article, I focus on one of the potential reasons for these problems: motivation. In fact, I argue that it is the main reason why several studies of learning and memory with *zebrafish* fail. Below, I detail some of the issues with motivating *zebrafish* in learning tasks. I do not provide a systematic review of animal motivation, as this broad and complex field has been discussed in comprehensive ways in entire books (e.g., [31,32,33]; also, see chapters in [34]). Instead, I provide selected experimental examples for why motivating *zebrafish* has been problematic, and I also discuss some of the solutions we have found to remedy the problems. I focus on the results and findings obtained in my own laboratory, while acknowledging that others have also made contributions in addressing related questions, for example, in the context of drugs of abuse [35] and emotional memory [36].

## 2. Food, a Ubiquitous Reward for Animals: Is the *Zebrafish* an Exception?

Before I delve into the details of why food can be a problematic reward in learning studies with the *zebrafish*, let me first define what is an appropriate unconditioned stimulus (US) and a conditioned stimulus (CS). Unconditioned stimuli are expected to induce an innate response, a response that does not have to be learned. In other words, unconditioned stimuli have inherent/instinctive value. This value comes from the fact that the animal’s genetic predispositions that have developed throughout the evolution of that species prepare the individual for responding to that stimulus even without having to have any prior experience with it. A conditioned stimulus, on the other hand, is expected not to have such inherent value. It is not expected to elicit appetitive or aversive responses if the animal has had no prior experience with that stimulus. In other words, the best CSs are those that are neutral, stimuli that the animal can perceive, yet do not reward or punish the animal.

Another general point I need to explain here concerns my focus on appetitive (rewarding) unconditioned stimuli. The reader will note that I excluded topics on aversive or pain-inducing USs. This focus is deliberate and have two main distinct reasons. One reason is that, in our hands, aversive conditioning has turned out to be more prone to the confounding effects of human handling than appetitive conditioning. In the latter, even when inappropriate human handling induces fear or anxiety responses (a topic I will discuss at the end of this perspective article), we found appetitive USs could work well because they counteracted the negative effects of human handling. In other words, we had a lot of room to change the behaviour of the fish (from anxious to habituated). With aversive USs, however, we faced a ceiling effect: human-handling-induced fear/anxiety responses were already high when we started conditioning, and these would have needed to be further enhanced by the punishment we employed as the US. Nevertheless, I also note that others have been able to employ aversive conditioning successfully, for example, in head-fixed *zebrafish* navigating a virtual environment [37,38], The other reason why we decided not to employ aversive conditioning concerns a mechanistic issue. Many aversive conditioning tasks with fish, including *zebrafish*, employ electric shocks as the US (e.g., [37,38,39]; also, see review in [40]). This is because the electric shock is easy to deliver in a temporally controlled and strength/amplitude-controlled manner. Such control is often more difficult with other aversive cues, including the smell or sight of natural predators [41,42,43,44]. Furthermore, results with the latter type of “natural” aversive cues have been found complex and contradictory in *zebrafish* studies (e.g., [40,41,45,46]). The controversies are likely due to a variety of factors that are often not controlled or not systematically manipulated in said studies, including human handling, stimulus strength, and/or aversive contexts. They may also be the result of the fact that *zebrafish*’s antipredatory (fear and anxiety) responses represent a broad behavioural repertoire, and depend upon the particular stimulus and the context in which they is presented (see, e.g., [47,48]). For these reasons, the *zebrafish* investigator often opts for the simple and easy-to-employ electric shock. However, the electric shock does not just induce pain. As it is employed in the water, the electric current reaches not only the sensory system of the fish but many other parts of its body too, including its brain. In the brain, the artificially injected current can produce the depolarization of neurons, trigger action potentials, and affect synaptic function in a variety of brain areas, circuits, and pathways. Briefly, for those who would like to study the mechanisms underlying learning and memory, such stimulus artefacts would be rather undesirable. The above issues are not a problem with appetitive cues. Food is one of the most frequently employed appetitive stimuli in learning studies conducted with laboratory species. Thus, I start my discussion with that stimulus.

Food is an obvious, and often employed, unconditioned stimulus. All species must obtain food in order to gain energy, building materials, and vitamins, all the components they need to build and sustain their body, and maintain their homeostasis. An individual that does not possess genetic predispositions to feel rewarded when eating food, to feel the need to seek out, obtain, and consume food, will have a major selective disadvantage. Thus, natural selection has led to the evolution of brains in which food induces the feeling of pleasure. In sum, food, as a stimulus, has gained inherent rewarding value, an evolutionary “trick”. There are numerous other unconditioned stimuli that have inherent values for a given species, some of which act as a reward (appetitive stimuli that induce positive or pleasure-related responses), others as a punishment (aversive stimuli that induce, e.g., avoidance or pain responses). However, food has been one of the best and most frequently employed USs in learning tasks with laboratory animals, due to the high evolutionary fitness value of this stimulus. Is the *zebrafish* different? Does food work well as a US in learning studies with *zebrafish*?

A review of the literature suggests that food, indeed, is a good reward for *zebrafish*. Learning studies, including those conducted in my laboratory as well, have demonstrated that the *zebrafish* is capable of acquiring the memory of association between food and a conditioned stimulus; i.e., food has turned out to be an appropriate US [21,23,24,27]. However, we also noticed that *zebrafish* almost always stopped performing in these tasks after a few trials. Occasionally, we were not even able to make the fish find the food, while, other times, the fish “quit” halfway through the training trials, i.e., before a robust-enough acquisition of CS–US associative memory has occurred. Via interactions with other *zebrafish* researchers, we learned that we were not alone. Many *zebrafish* studies suffered from the same issues, but these studies most often did not get published, likely because of the bias against negative results inherent in current publication practices [29,30]. While the general problem of a publication bias induced irreproducibility, and several other issues contributing to the replicability crisis in psychology, biology, and behavioural neuroscience have been thoroughly discussed both for the *zebrafish* [30] and for the most frequently employed biomedical research organism, the house mouse [29], specific solutions for the failed learning studies with *zebrafish* remained elusive.

Here, focusing on the question of motivation in *zebrafish*, first, I will consider an issue that has not been properly discussed in the literature: the potential problems with food as a reinforcer in learning studies with *zebrafish*. Subsequently, I extend my discussion to other unconditioned stimuli, i.e., to the question of how to motivate *zebrafish* with USs other than food. Last, I also discuss a factor that may interfere with the motivation of *zebrafish* to learn in associative tasks, the aforementioned fear and anxiety responses induced by human handling.

## 3. *Zebrafish* in Nature: How and What Do They Eat?

To properly appreciate the issue surrounding food as an unconditioned stimulus, consider how *zebrafish* forage in nature. Before going into specifics, let me address a general question: why should we care about nature, i.e., the ethology and ecology of the studied species, the *zebrafish,* in this case? This question has been discussed in the literature multiple times, including by us [49,50,51]. Although not all agree, the main points are as follow: Despite the millennia- (in the case of the mouse) or decades-long (in the case of the *zebrafish*) laboratory breeding, laboratory organisms maintain a footprint of their evolutionary past. Although there may be genetic changes due to unintended or intentional artificial selection, and/or to random genetic drift (the random fixation of alleles due to the finite effective population size of the animals in the laboratory), the fundamental features of the biology of the studied species remain. These include the anatomical and neurobiological, as well as behavioural characteristics of the studied organism.

**Figure 1:**
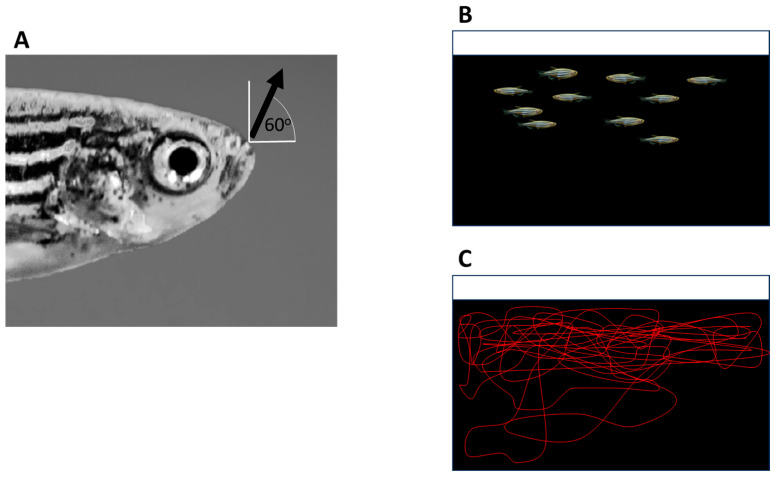
*Zebrafish* swim and forage near the surface of the water. Panel (**A**): The anatomy of the mouth suggests the *zebrafish* is adapted to capture small food items (insects) near or on the surface of the water, a foraging strategy observed in nature as well as in the laboratory (see text). The arrow shows the angle of opening of the mouth (approximately 60o to the horizontal plane). It also illustrates the expected (upward) direction of the prey-capture strike (indicated by the arrowhead). Panel (**B**): Illustration of the positioning of a shoal (group) of habituated *zebrafish* swimming in the middle to the upper water column (note that, under aversive conditions or in response to fear-inducing stimuli, *zebrafish* tend to stay close to the bottom; for further details, see text). Panel (**C**). Swim path of a single *zebrafish* habituated to its aquarium recorded during a 5 min observation session by video-tracking software Ethovision Color Pro version 16 (Noldus Info Tech, Wageningen, The Netherlands) (Gerlai unpublished data). Note that, although the subject explored the lower part of its aquarium, it tended to swim in the middle to upper level of the water column the majority of the time.

**Figure 2:**
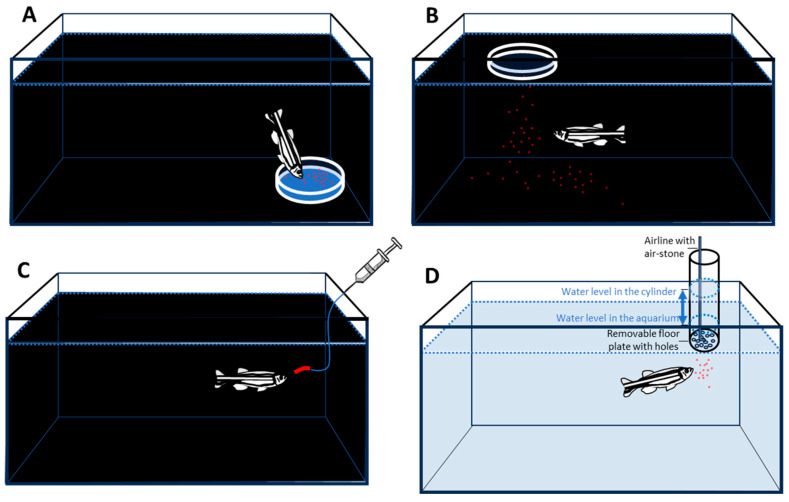
Examples of different food delivery methods. Panel (**A**): Food is presented in a tray on the bottom of the tank. This method allows excellent localization of food. However, the disadvantage of this method is that feeding from the bottom is an ethologically inappropriate and unnatural foraging strategy for the *zebrafish*. Another disadvantage of this method is that it presents all the food at once. This poses a problem. If only a small amount of food is presented, it is consumed quickly and no food remains (i.e., no US is present) for the rest of the training trial. If large amount of food is presented, the fish may be satiated and thus may not continue the training trial. Panel (**B**): Food is presented inside a floating ring on the surface of the water. This presentation method observes the natural behaviour of *zebrafish* (feeding from the surface of the water; i.e., it is ethologically relevant). However, similarly to the method shown in panel (**A**), it suffers from the issue of inability to achieve an optimal food delivery amount that remains unchanged throughout the training trial. That is, all food is presented at once and is available from the beginning of the trial, and temporal control of spaced-food delivery cannot be achieved. This method of food delivery also suffers from another disadvantage: numerous food types (including some pellets, all flakes, and all live food) when delivered in this manner, cannot be properly localized, but, instead, the food particles will disperse. Localization of food (the US) near the CS is crucial for CS–US association conditioning trials. Panel (**C**): Food is presented using a syringe. This method is excellent in terms of its ability to control the location and the amount of food presented to the fish. If a peristaltic pump connected to the food delivery tube is employed instead of a syringe, the method can also achieve a relatively continuous (constant) food delivery rate throughout the training session. This method is usually employed with gelatinous food substances, which can be extruded from the tube. The disadvantage of the latter, however, is that such food substances are bulky. The bulkiness of the food item is problematic, as *zebrafish* are not adapted to such food items in nature. Moreover, the bulky food can quickly satiate the fish and lead to its reduced motivation to continue the CS–US association training trial. Panel (**D**): A recently developed method with the use of a food delivery cylinder that attempted to address the issues of previously employed food delivery methods in learning tasks with *zebrafish*. This method uses a cylinder filled with a food solution (water in which food particles are dispersed). The water level in this cylinder is above that of the test tank (indicated on the figure), which creates gravitational pressure (blue double-headed arrow). The height of the water column can be adjusted by moving the cylinder up or down. This pressure forces the food particles to exit through the holes of the floor plate of the cylinder (indicated on the figure). The floor plate can be exchanged to accommodate different food particle sizes. Inside the cylinder, an airline/air-stone ascertains that food particles remain afloat and evenly distributed. This method has been shown to allow stable and continuous delivery of small amount of food in a temporally controlled manner throughout 5 min long periods, length of time often employed in CS–US associative training trials for *zebrafish* (for further details on this method and discussion on other previously employed food delivery methods for *zebrafish*, see [25]). The apparatus was recently used to determine preference for certain food types in binary choice tasks with *zebrafish* [59]. Panel (**D**) is adapted from [59].

For example, a *zebrafish* whose ancestors have been bred in captivity over a hundred generations will still show the anatomy of the mouth as pointing upward. This anatomy has been interpreted, and confirmed as such, as an indication of the foraging strategy of that fish species: an upward-pointing mouth means a specialization to catch food items from the water surface or close to the surface, perhaps in midwater, but definitely not from the bottom (for a review and expanded discussion on this topic, see [20]; also, see Figure 1A). Although only very few have studied the natural behaviour and habitats of *zebrafish* [52,53,54], this is what has been observed as the foraging strategy of this species in the wild as well as in the laboratory [20,55]. Supporting this is also the finding that showed habituated *zebrafish* spending most of their time swimming near the surface or in midwater, but not near the bottom, of experimental tanks (e.g., [56]; also, see Figure 1B,C). Furthermore, in nature, *zebrafish* have been found to consume small terrestrial insects that fall into the water as well as small aquatic organisms that float in the water [55]. These prey items are available throughout the day and are not concentrated in large numbers or amounts in specific locations, but, rather, tend to be spatially distributed. In accordance with this, *zebrafish* are expected to eat throughout the day (it is a diurnal species that sleeps during the night), and are expected to eat small amounts of food at a time. However, learning paradigms do not employ food as the US according to these features of the *zebrafish*. Often, a bulky food item (e.g., gelly-belly, Florida Aqua Farms, Dade City, FL, USA) is used [26,57,58], or full access to a large amount of food, presented as US, is given to the experimental animal, instead of the temporally controlled continuous or repeated delivery of small amounts at a time (see the learning studies with *zebrafish* reviewed in [18,19]). Another issue with the food employed in learning tasks is that it is often placed on or near the bottom of the test apparatus (an illustration of some of the above problems is given in Figure 2A–C).

Lastly, the food types employed are often rather artificial: they include manufactured flakes, pellets, or gelatinous substances, whose size, shape, and, perhaps, taste do not resemble the characteristics of food *zebrafish* encounter in nature. Could these artificial aspects of the food reward affect the motivational value of this US and, therefore, the learning performance of *Zebrafish*? The answer is a likely yes, a topic I will explore below.

## 4. The Artificial Nature of Food Reward in Learning Tasks, and What We Can Do About It

In a food-reinforced learning paradigm using a plus-shaped maze, we demonstrated that *zebrafish* were capable of acquiring CS–US (elemental) associative memory after 20 trials during which the CS and US were co-presented [27]. In the same study, using another group of *zebrafish*, we could also demonstrate that *zebrafish* were capable of learning the spatial location of the US; i.e., they could acquire spatial (configural or relational) memory [27]. The US we employed in both the elemental and the configural version of the task was gelly-belly, a gelatinous fish food that was placed in syringes. The food was delivered from these syringes at a particular location of the plus-maze. Although the study was a success, subsequent follow-up investigations (unpublished) revealed issues. For example, we wanted to investigate whether memory performance could be further strengthened by conducting additional training trials. We also wanted to see how long *zebrafish* remembered the acquired memory and whether they needed intermittent training trials to extend their memory span. However, none of these follow-up studies were successful. *zebrafish* started to ignore the food reward: the fish stopped eating it and also stopped responding to stimuli (the CS or the spatial location) that would predict the presence of food. Concurrent with this, we also noticed that the fish substantially reduced their intake of their regular food, i.e., the food they would receive in their home tanks (unpublished results). We speculated that these failures could be the result of *zebrafish* becoming satiated with gelly-belly. Another possibility we considered was the location of the delivery of the gelly-belly: it was delivered near the bottom of the maze, and *zebrafish* had to nibble at the food protruding from the syringe, an unnatural foraging strategy for the *zebrafish*.

In an attempt to improve the above problematic aspects of food as a US, in another learning task [24], we decided to use commercially available dried aquarium fish food, tetra-min flakes, as the US. The advantage of this food compared to gelly-belly, we theorized, was that it floated, at least for a while, on the water surface. Furthermore, its particle size could be reduced by crushing the flakes, thereby mimicking small insects that fall into or float in the water. This study also showed the development of significant CS–US associative memory after 20 training trials [24], and the fish appeared not to be satiated with this food; i.e., they continued to seek and consume it even after the completion of the 20 training trials (unpublished results). However, we encountered another problem with this food type. Although the flakes remained on the surface for about a minute, as they became saturated with water, they started to fall, and the movement of the fish started to disperse them (Figure 2B). Thus, a proportion of food ended up on the bottom of the training tank and often several cm’s away from the intended location. Localizing the food precisely next to the CS or at a precise spatial location was thus not possible. Furthermore, all the food was presented at once, i.e., from the beginning of the trial, and could be consumed by the fish at once. That is, prolonged and temporally controlled food delivery could not be achieved.

At this point, we realized that a systematic analysis of food types and their delivery method is needed to optimize food as a US for appetitive learning tasks. Another potential solution we decided to explore was the identification of alternative reinforcers for appetitive associative conditioning. I will discuss the former topic first, i.e., our efforts to make food work well in appetitive conditioning tasks for *zebrafish*, and subsequently discuss the latter, i.e., potential alternative USs we have identified.

## 5. What Do *Zebrafish* Like to Eat, and How Should We Deliver This Food: A Proof-of-Concept Analysis

A lot is known about the nutritional requirements of *zebrafish*, and several commercially available food items have been in use in *zebrafish* facilities across the world. However, the choice of what is fed to *zebrafish* is often based upon anecdotes, tradition, or just practicality, and is made by the human experimenter, not the fish. Facility managers may choose the food type based upon what is available in aquarium stores or from commercial vendors. Researchers may develop a “sense” for what type of food *zebrafish* are most “enthusiastic” about. Others may worry about what type of food has the highest nutritional value, and observe what food should be offered to achieve the fastest growth. Nevertheless, rarely has the question been asked: what do *zebrafish* actually prefer? This question is particularly relevant in learning studies, where food is used as a US. If the food item is highly preferred by *zebrafish*, it is expected to serve as a stronger motivator to learn. Thus, we conducted a proof-of-principle study to demonstrate that *zebrafish* do show clear food-type preferences. For this, we developed a novel food delivery system [59] (also, see Figure 2D). The features of this system were designed with learning and memory testing in mind. The hardware was simple. Food was dispersed in an acrylic cylinder filled with water. This cylinder was positioned so that its water line was several cm above that of the experimental tank. This created a gravitational water pressure that pushed the food items floating inside the cylinder out into the test tank via small holes on the bottom of the cylinder. Notably, the hole-plate of the cylinder could be exchanged so that differently sized holes could accommodate different food types (Figure 2D). The height of the water column, the outflow pressure, could also be modified by moving the cylinder up or down relative to the water-line of the test tank. Lastly, inside the cylinder, a small air tube allowed us to deliver bubbles, which would keep the food items floating and evenly distributed. This simple hardware design, after a lengthy dry run experimenting with the strength of the bubbling action, size of holes, and amount of gravitational pressure, allowed us to achieve the localized and continuous delivery of three different food items near the water surface of the test tank [59]. We chose to work with three food types based upon their frequent use in *zebrafish* facilities: Zeigler *zebrafish* pellets (Zeigler), tetramin flakes (Tetra, Germany), and nauplii (larvae) of the brine shrimp (*Artemia salina*) (San Francisco Brand). I note that brine shrimp nauplii live in high-salinity water but also survive for hours in low salinity, and thus remain mobile for the duration of food delivery and experimental procedures employed in *zebrafish* learning studies. Details of the specifications of the hardware design and the procedure employed are given in [59]. Briefly, in this particular application, we employed different water column heights depending on the food type. That is, for flakes, pellets, and artemia nauplii, we used 4, 6, and 12 cm water columns above the water level of the experimental tank, respectively. Furthermore, we dispersed 35, 65, and 60 mg total weight of food for flakes, pellets, and artemia nauplii, respectively, and we employed the same floor plate with 1 mm holes for all these food types [59]. Once we were satisfied with the hardware design and procedural details of the setup, we conducted binary choice tasks with *zebrafish* using the above food items. That is, we presented two of the food types at a time in all possible combinations, a total of three binary choice tasks. The most important result of this study was that *zebrafish* showed a clear preference [59]. They preferred artemia to both pellets and the flakes, and chose roughly equally when these latter two were contrasted (co-presented) [59]. These conclusions were drawn based upon measuring the duration of time the fish spent in the vicinity of one versus the other food delivery cylinder, the distance between the fish and one versus the other feeding cylinder, and the number of times the fish visited one versus the other cylinder. Furthermore, an analysis of the temporal trajectories of the values of the above behavioural parameters also demonstrated that the behavioural responses of the fish to these food types were stable across the 5-minute-long food preference test. This was partly because, as we observed, the food delivery cylinders were able to deliver their respective food items continually and consistently, at least for this period of time. As 5 min is often the length of *zebrafish* training trials, we concluded that the newly designed hardware and food delivery procedure should be appropriate for learning studies; i.e., food delivered using this method could serve as a US. Whether this novel food delivery method will indeed improve the ability of the investigator to conduct food-reinforced learning tasks with *zebrafish* will need to be empirically explored. Nevertheless, as the amount and speed of food delivery can be controlled using the above-described novel method by setting how much food is placed in the cylinder and how much gravitational pressure is employed, I also argue that this method will likely enable investigators to avoid the potential issue of satiation during training trials. The latter point I will further explore below.

## 6. Food Satiation, and How to Avoid It in Learning Tasks with *Zebrafish*, a Paradoxical Discovery

As mentioned before, in our hands, satiation with food has been a major problem with the *zebrafish* we trained in learning tasks. There are several ways satiation with food has been addressed, or, in general, an increase in the rewarding value of food has been achieved, in animal research. The most frequently employed solution has been food deprivation. For example, rodents are usually chronically deprived of food before the associative training trials are started (e.g., [60,61]). This method seems logical. Hungrier animals should be more motivated to seek out and eat food, and thus should learn better in food-reinforced learning tasks. This assumption, however, did not hold for *zebrafish*. The discovery came in a serendipitous manner.

Although some feed *zebrafish* twice or three times a day, several *zebrafish* facilities feed *zebrafish* only once a day, a feeding regimen that does not seem to have deleterious consequences but saves time. In our facility, we usually feed adult *zebrafish* 2–3 times per day, but, during the pandemic, we could not continue this practice. Due to human-health-and-safety-related restrictions, we had to minimize the feeding frequency to once per day. Research during this period was also severely impacted by pandemic-related restrictions, but did not stop. One of the research studies that had to be continued was a food-reinforced learning study. However, the study failed. Experimental *zebrafish* did not appear to be interested in eating the food presented to them as the US. They were unmotivated, and thus did not learn the association between CS and US. The unexpected result prompted us to revisit my prior personal observations I had made with a variety of fish species. For example, in our tropical fish tanks, when fish were fed more frequently and with more food, they appeared to become vigorous, they appeared to be wanting to eat more, they followed the person who was about to feed them, and they congregated and became active near the feeding location, whereas, when we did not feed the fish frequently, they appeared to show the opposite response—they slowed down, and started to be less interested in obtaining food. Nevertheless, these were just personal observations, anecdotical evidence with no statistically significant results/data documenting this potential feeding effect. Thus, to test the possibility that increasing the feeding frequency and food amount may actually increase the motivation to eat the food in *zebrafish*, we decided to conduct a simple experiment [20].

*Zebrafish* were randomly divided into two experimental groups [20]. In the first group, fish were fed once a day, but, in the other, they were fed five times a day (for all fish, each time, with the same set amount of food) for three months. Subsequently, the fish were trained in a CS–US association trial in which the CS was a colour cue and the US was the same food type they received during the past 3 months. The experiment yielded unequivocal results. The *zebrafish* that were fed 5 times a day consumed dramatically more food during all 12 training trials compared to fish that received food only once a day prior to these trials [20]. I emphasize that, during the training trials, the amount of food, the location of food delivery, the experimental apparatus, water chemistry, and all other experimental and procedural factors were identical between the two groups of fish, and all fish were trained and tested in a randomized order with respect to their prior feeding condition. Subsequent to the training trials, the fish were further divided into two groups. In one, food would be paired with a CS (CS–US conditioning), the so-called paired group, and, in the other, the CS and US would be presented in a random manner (unpaired group). The expectation with this experimental design was that fish in the paired group should learn and remember the association between CS and US and, thus, should show a strong preference for the CS at a memory probe trial, whereas fish in the unpaired group should not be able to learn the association and should not show any preference for the CS at the memory probe trial. This is exactly what was found, but only for the 5-times-a-day fed fish [20]. These fish were motivated to find the food and they associated the CS–US, and thus could show excellent memory of the CS when that cue was presented alone. However, the once-a-day-fed fish could not [20]. This is a paradoxical finding that suggests the *zebrafish* functions the opposite way to rodents. A chronic increase in feeding does not diminish the motivation (hunger) to eat food, but actually increases it. How is this possible?

The answer is speculative, as, at this point, we do not have clear physiological or ecological evidence for the argument I am about to make. Nevertheless, consider the following. The freshwater streams and ponds where *zebrafish* live in India and Nepal, the natural habitats of this species, experience a biphasic annual cycle, the monsoon and the dry season. During the former, a lot of debris and organic waste enters the water, the temperature slightly increases, and aquatic and terrestrial insects and crustaceans become more abundant. *zebrafish* have been adapted to this environment and, I argue, may be able to utilize the increased abundance of available food during the monsoon season. This is the season when they likely are more active and when they spawn more frequently, and, in general, this is when they are expected to increase their metabolism. During the dry season, they are expected to slow their activity and metabolism to correlate these biological/physiological changes with less abundant food. These are responses that are opposite of what one would expect for the homothermic rodent that must maintain its metabolism and body temperature within a narrow homeostatic range. In other words, I argue that an abundance of food accelerates the foraging activity of *zebrafish*; i.e., it likely sets both the motivation to obtain food and correlated metabolic activity higher, whereas a chronically reduced food amount diminishes these processes. Irrespective of whether this argument is correct or not, our results demonstrated that a chronically increased frequency of feeding, and, with it, the increased total daily food intake, will enhance *zebrafish*’s motivation to work for food in CS–US conditioning trials, and will improve learning and memory performance [20]. I note that the effects of the feeding status on behavioural responses have also been explored in larval *zebrafish*, and hunger was found to shift avoidance to an approach of potentially dangerous (predatory) stimuli in the 7-day post-fertilization old larvae [62].

Given the above-described issues with food, and given that systematic studies have not been conducted to address them, we also started to explore alternative motivators we could use as US in associative learning studies with *zebrafish*. Next, I will review some of these studies.

## 7. Food Is Not the Only Possibility: Alternative Motivators (USs) for Learning Studies with *Zebrafish*

**Figure 3:**
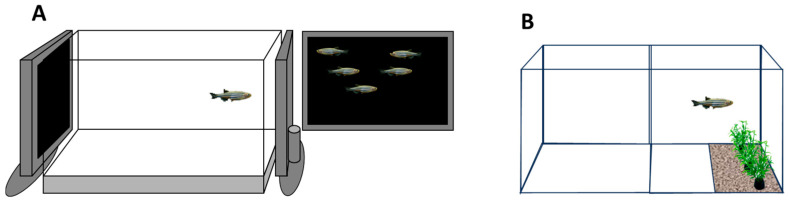
Examples of appetitive unconditioned stimuli (USs) as alternatives for food reward. Panel (**A**): Sight of conspecifics. The experimental tank with a single experimental fish in the tank flanked by stimulus presentation screens is shown. An example of animated images of conspecifics presented on the screen is shown on the right side of the tank. Sight of conspecifics has been found to induce a robust shoaling response (approach of the stimulus and subsequent staying in the proximity of the stimulus) in single experimental *zebrafish* (e.g., [65,66]). The stimulus can be live *zebrafish* placed outside of the experimental tank or animated images of *zebrafish* (as shown in this figure) [67]. The sight of conspecifics has been shown to serve as an excellent appetitive US in associative learning tasks with *zebrafish* (e.g., [28]). Panel (**B**): Presence of naturalistic cues mimicking gravel bottom and aquatic vegetation. These cues borrowed from nature have been found to be good motivators (rewarding US) in appetitive associative conditioning with *zebrafish* [68]. Adapted from [65,68].

The *zebrafish* is a shoaling species. Both in nature and in the laboratory, these fish are found to form groups in which group members maintain a fairly steady distance from each other (reviewed in [63,64]). We developed a simple paradigm to demonstrate and also to quantitatively assess the strength of shoaling in *zebrafish* [65] (Figure 3A).

A single fish placed in a 40-litre glass tank would respond to the sight of conspecifics presented on the side of this tank by immediately swimming to that side and staying on that side (the shoaling response), approximately 5–10 cm away from the presented shoaling stimulus (e.g., [65]). We also found that, once the shoaling stimulus was removed, the single *zebrafish* would return to the middle of the tank; i.e., it would stop showing a preference for any one side. In fact, we discovered that one could keep presenting and removing the shoaling stimulus repeatedly and the single test fish would respond consistently; i.e., the shoaling response did not habituate [66]. We also analyzed how different methods of stimulus presentation may affect the responses of the test fish [67]. For example, we presented live stimulus *zebrafish* in the test tank (visual, olfactory, auditory, and lateral line cues were, thus, all present, and the stimulus fish could interact with the test fish), or live *zebrafish* outside the test tank (only visual cues were present and the stimulus fish could interact with the test fish), or a playback of live stimulus fish video-recorded previously (visual cues were present but the stimulus fish could not interact with the test fish), or animated images of *zebrafish* (visual cues were present, and the stimulus fish could not interact with the test fish and also swam in 2D) [67]. We found that these different methods induced a statistically indistinguishable shoaling response, which was equally robust for all these differently presented social stimuli. We also conducted another study in which we asked what aspects/features of the conspecific image may be most important to *zebrafish* [69]. We investigated if the speed, size, body proportions, location in the water column, colour, and pattern may be important cues to which *zebrafish* attend. We found that, as long as the image was moving with a realistic speed in the middle and upper water column and was not abnormally elongated relative to a natural-looking *zebrafish*, the image induced a robust shoaling response (e.g., [69]). Thus, we concluded that an image that looks and moves similarly to a live *zebrafish* should be a stimulus that induces shoaling responses in the test subject, a conclusion that was later confirmed by others (e.g., [70]). Thus, the above results suggested to us that the sight of conspecifics may be an excellent US for learning studies. Indeed, this was what we demonstrated using a plus-shaped maze, in which the presence of conspecifics (placed into a stimulus tank outside at the end of one of the arms of the plus maze) was paired with a visible cue, a red cue card [28]. Once more, we ran two groups of fish, a paired group (for which the CS (red cue) and the US (conspecifics) were co-presented), and an unpaired group (for which the CS and US were presented randomly). As expected, fish of the paired group would show a significant preference for the red cue card during a memory probe trial (when only this cue, i.e., the CS, was present and the conspecifics, i.e., the US, were absent) [28], whereas the unpaired group of fish ran randomly, i.e., showed no preference for the red cue. Subsequent learning studies with *zebrafish* in our laboratory also successfully utilized this US and, for example, could demonstrate the concurrent acquisition of elemental (CS–US) memory and configural/spatial memory (relationships among multiple CS–US association) [25]. We have also discovered that the sight of conspecifics induces the rapid elevation of dopamine and DOPAC levels in the brain of *zebrafish*, a response that is selective both in terms of the effect on this specific neurotransmitter system (but not on others) and also in terms of the stimulus that can induce it [71]. Last, we demonstrated that the pharmacological disruption of dopaminergic neurotransmission (via blocking the postsynaptic D1-dopamine neurotransmitter receptor) dose dependently impairs or abolishes the shoaling response in *zebrafish* [72]. Thus, we concluded that the sight of conspecifics is rewarding, and that the behavioural response to conspecifics is mediated by the dopaminergic neurotransmitter system.

At the time of this discovery, we were (and still are) exploring the effects of acute, chronic, and embryonic ethanol exposure on *zebrafish*. We found that shoaling is severely disrupted by alcohol administration in all three of these regimes [65,73,74,75,76,77]. We also found that learning and memory were also disrupted by alcohol [24,78]. However, we could not distinguish whether alcohol disrupted learning and memory because of its effects on motivation (shoaling) or via another psychological/neurobiological mechanism. From a mechanistic standpoint as well, the dissociation of alcohol effects on these two processes (motivation/shoaling vs. learning and memory/neuronal plasticity) was required, because neuronal plasticity subserves multiple behavioural phenomena, including learning and memory, and also social cognition and social behaviour. The above is true for a variety of other drugs and, also, genetic manipulations that affect neuroplasticity. Thus, we realized, if we want to study the mechanisms of learning and memory in *zebrafish*, we may need other motivators, USs that do not utilize shoaling. In this last paragraph of this segment, I describe one such motivator: cues from nature, or, as we call them, “natural stimuli”.

Our philosophy has been to learn and borrow from nature [49,50,51]. We argued that, as *zebrafish* must have adapted to its natural environment, it should innately “recognize” certain elements of, or cues in, their natural habitat. Thus, we started exploring whether *zebrafish* exhibits an innate preference for certain features/cues in its environment. In one experiment, for example, we presented *zebrafish* with a choice: a barren side of its test tank or a side where a photograph of a naturalistic gravel was affixed to the bottom glass and plastic plants mimicking aquatic vegetation of a small tropical freshwater stream of India was placed [68] (also see Figure 3B). The study showed that *zebrafish* exhibited a clear choice: they preferred the side where these “natural stimuli” were placed [68]. The next step was easy: these natural stimuli (US) were co-presented with a neutral stimulus, a red background (CS) for the paired group, and were presented randomly for the unpaired group. After a few training trials, the *zebrafish* of the paired group showed a clear preference for the red background at a probe trial in which the natural stimuli were absent, whereas the unpaired group ran randomly [68]. Thus, the natural stimuli worked as a US! They motivated the *zebrafish* to learn and remember the CS–US association. What was even more remarkable was that the fish could successfully acquire this CS–US associative memory after only four training trials, the fastest memory acquisition speed we have seen in our laboratory with *zebrafish* [68].

Although successfully employed, the above naturalistic cues suffer from a problem. We do not know what aspects/features characterizing these cues *zebrafish* attend to and/or prefer. This problem is not unique to the above naturalistic cues, as most stimuli employed in behavioural studies with *zebrafish* have not been systematically investigated to answer this question. The scientist may find that *zebrafish* significantly prefer (appetitive) or avoid (aversive) cues, but will often not know what aspects of these complex “compound” cues induced these effects. Briefly, systematic studies are needed to analyze the components of these compound-cues, akin to the one conducted with *zebrafish* images [69], to discover such aspects, i.e., the minimum number and the kinds of components of the naturalistic stimuli that may induce maximal appetitive (or aversive) responses in *zebrafish*.

The last point I consider in this section is the question of olfactory cues. I have focused on visual cues in most discussions in this review so far, and, indeed, also in my own research with *zebrafish*. This focus has a conceptual and a practical reason. The conceptual one is that *zebrafish* is a diurnal species with excellent vision, and it has been shown to respond strongly to cues of this modality [47,48,67,79,80]. The practical reason is that visual cues are easy to control, record, deliver, and remove, using simple consumer-grade devices, including video-cameras, computers, computer monitors, and LEDs. Nevertheless, *zebrafish* in nature, and in the laboratory as well, perceive cues of other modalities as well. One such modality, olfaction, may be worth mentioning briefly here (for a general review of the effects of chemosensory cues on the behaviour of fish, see [81]). Although the onset and offset of olfactory cues may be more difficult to control in a temporally and spatially precise manner, and although devices that could deliver and/or remove such cues may require some ingenious engineering, they have already been successfully employed in *zebrafish* research. In this review, I have already mentioned alarm substances, including the synthetic substance hypoxanthine 3-N-oxide, which have been shown to induce robust fear and/or anxiety responses in *zebrafish* [42,44]. However, appetitive chemical cues have also been identified for *zebrafish* in the context of associative learning. For example, certain amino acids have been shown to induce appetitive behaviours in *zebrafish*, and have been found to be appropriate as unconditioned stimuli in learning tasks for this species [82]. Furthermore, olfactory cues have also been successfully used as conditioned stimuli in associative learning tasks for *zebrafish* [83,84]. Thus, in the quest to find proper unconditioned as well as conditioned stimuli for the analysis of learning and memory in *zebrafish*, olfactory cues should also be considered.

## 8. Fear: A Significant Motivational Confound in Appetitive Learning and Memory Tasks with *Zebrafish*

The last topic I consider in this review is fear and/or anxiety, an important motivator that may represent a significant confound for appetitive (reward-based) learning tasks with *zebrafish* and with other laboratory species as well. Here, I use the terms fear and anxiety in an interchangeable way, as the manifestation of (that is the behavioural responses associated with) these two behavioural/psychological states greatly overlap in *zebrafish* [45,46]. Nevertheless, I note that fear is usually defined operationally as responses to the appearance of aversive stimuli, i.e., to imminent danger, whereas anxiety is usually defined as responses to aversive contexts without clear signs of imminent danger. The latter is also often defined as a more prolonged, chronic response, whereas the former is defined as a more acute response. Perhaps the best demonstration of how fear/anxiety may interfere with experimental results is the study by Crabbe et al. [85] conducted with mice. The study, which was published in Science, created quite a stir. The goal of these authors was to investigate how reproducible behavioural results were with mice. The three authors of the study coordinated their work and made sure that all aspects of their experiments were identical. For example, the pieces of equipment they used were the same, the procedures were the same, the age of their mice was the same, and the inbred and null mutant mouse strains were the same across the three laboratories. Furthermore, all experiments were conducted concurrently, i.e., exactly at the same time and in the same order in the three laboratories. The authors demonstrated that the relative differences among inbred and mutant mouse strains were remarkably similar across the three laboratories [85]. However, there were some notable exceptions to these similarities. While most behavioural tests did provide consistent rank orders among the studied mouse strains across the three laboratories, some tests did not. Instead, these behavioural tests yielded laboratory-specific idiosyncratic results. Wahlsten et al. [86] provided a more detailed analysis and in-depth discussion of the results, and identified several factors that may have contributed to the laboratory-specific differences in the results. One of these factors was an unexpected common feature among the unreliable behavioural tests. These tests all involved a substantial amount of human handling, and were all dependent on, or influenced by, fear and anxiety responses. Retrospectively, the investigators realized that there were substantial differences among the technicians who handled the mice [86]. That is, unexpected differences in human handling led to differential, laboratory-specific fear and anxiety responses in the mice, which interfered with their performance in certain behavioural tests. The bottom line of this story for us is that human handling is hard to standardize.

In my laboratory, failures with learning studies with *zebrafish* were also often due to elevated or variable fear and anxiety responses. In some experiments, the fish appeared habituated and performed very well, while, in others, they failed to complete their CS–US association training (inter-experiment variability); yet, in other experiments, some fish appeared habituated, while others were highly anxious (intra-experiment variability). Unfortunately, these failures did not receive enough attention in the literature, a problem to which we also contributed by not being able to publish our failed studies [30]. Nevertheless, to help discover the causes underlying the above problem, we characterized the fear and anxiety responses of *zebrafish*, and also investigated the different stimuli and contexts that may trigger such responses [41,45,46]. What we learned from these studies is that *zebrafish* have a complex anti-predatory behavioural repertoire, behavioural responses that are specific to the nature of the aversive stimuli or the context in which they are employed [47,48,87,88,89]. A thorough review of these results is beyond the scope of this paper. Nevertheless, a few examples of typical fear/anxiety responses we have seen in our learning studies are worth mentioning.

Passive avoidance is one anti-predatory strategy we have seen in *zebrafish*, especially at the early phases of training in learning studies. A fish exhibiting this type of response could freeze for extended periods of time, and thus would not navigate their maze and could not find a spatial location or a CS with which a US was paired. Another immediate response to aversive stimuli (e.g., human handling) is a set of panic reactions, including erratic movement. Fish that exhibited this response tended to have no interest in foraging, or in obtaining any other sorts of reward, and thus could not learn the association between food, or other rewarding US, and the CS. Yet, other fish performed what we call “thrashing”, i.e., forceful swimming perpendicular to the transparent wall of their test tank, an active avoidance response that gives the impression of a fish trying to escape from the test tank. Fish exhibiting such responses could not be efficiently trained to associate the CS and the appetitive US either. Clearly, in these cases fear/anxiety interfered with the behavioural performance of the experimental *zebrafish* in the learning task. In aversive conditioning, i.e., in tasks where the US is a punishment, the fear/anxiety induced by human handling may be even more problematic as it may create a ceiling effect, high fear that may not be further enhanced by aversive US. Nevertheless, in both aversive and appetitive conditioning, human-handling-induced fear/anxiety is expected to induce error variation because the handling of *zebrafish* is hard to standardize, perhaps even harder than with mice [85].

How can fear/anxiety, human-handling-induced or otherwise, be avoided or minimized? Unfortunately, this question remains mainly unaddressed for *zebrafish* studies. Nevertheless, below, I will discuss some potential solutions and our different attempts that already yielded promising preliminary results.

The first potential solution I consider here is to habituate the fish to the test tank and test procedure. Most learning studies require a specific experimental tank. This could be a simple glass tank equipped with stimulus delivery apparatu, or an acrylic maze, including +-shaped or Y-shaped mazes, for example. Either way, the test environment is novel to the experimental fish. Novelty has been known to be aversive to *zebrafish* (e.g., [56,89], and references therein). Furthermore, most learning studies are conducted with single fish placed into the test apparatus. The *zebrafish* is a social, shoaling species, and individuals prefer staying in close proximity to each other [63,64]. Thus, the novel nature of the test apparatus coupled with the experimental fish being alone in that tank is expected to be aversive. The experimental procedure is also expected to induce fear and anxiety, as it involves human handling, catching the fish with a net and transporting the fish from its home tank to the test tank. Thus, we have developed elaborate habituation procedures to make the fish get used to the above fear/anxiety-inducing aspects of our learning studies [26,27]. For example, we employed habituation trials that were expected to change sequentially from less to more aversive. That is, initially, we placed multiple fish in the test tank, and allowed them to explore the tank together. On subsequent habituation trials, we reduced the number of fish placed in the test tank at a time in a step-wise manner until we reached the desired one-fish-per-test-tank density [27]. Our attempts to habituate the experimental fish this way often succeeded, but not always. Occasionally, we found the opposite result: instead of habituation, the experimental fish became sensitized, and exhibited even more robust fear and anxiety responses than before the habituation trials were started (unpublished observations). We realized that the issue, just like with mice [85,86], was the difficulty controlling human handling itself. If not conducted very carefully, repeated human handling performed during the intended habituation trials led to sensitization and elevated fear and anxiety in the fish. This was the point when we turned our attention to the procedure of human handling itself.

We decided to compare different human handling procedures to investigate which one(s) was/were less invasive, i.e., less fear/anxiety-inducing [80]. The human handling of *zebrafish* usually involves netting the fish. We considered many aspects of this handling procedure, including the length of time during which the fish were chased by the net and how vigorous this chasing was. We also considered the amount of time during which the fish were lifted out of the water (the length of exposure to atmospheric air). Our preliminary observations suggested large differences in how our students performed netting in terms of the above aspects, which we suspected might have been behind the increased variability of fear/anxiety responses experimental fish in our learning studies exhibited. Thus, in a systematic study, we compared four different handling procedures [90]. One mimicked what inexperienced students may do, i.e., students who would move the net fast and would chase the target fish vigorously for longer periods of time. In another handling procedure, the net was moved slowly and for a shorter period of time, representing a more careful approach to catching the fish. The third handling method was one which was rarely used in *zebrafish* facilities. This method included gentle netting as in the second handling method described above, but the period of air exposure was minimized by placing the fish (while still in the net) into a beaker containing the same system water as that of the home tank of the fish. This method thus minimized both net-chasing and air exposure. The fourth method employed no netting and no air exposure at all. This method, called pouring, involved slowly siphoning off about 2/3 of the home tank water, then moving the fish in the home tank to the test tank, and pouring the fish from the home tank into the test tank. I note that the different temporal aspects of the above four procedures were strictly controlled and the four handling methods were employed in a randomized order. The results unequivocally showed that the traditional netting procedure, especially when it was mimicking what an inexperienced researcher would do, was the most invasive, and most fear/anxiety-inducing [90]. On the other hand, the pouring method was the least invasive. The beaker method was almost as good as the pouring method in terms of not inducing fear/anxiety responses [90]. I also note that, as the pouring method is difficult to employ when one wants to select a single fish at a time for CS–US association training, we subsequently used the beaker method for our learning experiments with success. Lastly, I emphasize that the above study [90] investigated the effects of acute human handling, and stress that we do not yet have empirical data on how the different handling methods may affect the behaviour of *zebrafish* when employed chronically.

Despite being less invasive, the beaker method still involves netting (catching the fish with short air exposures), transporting the fish, and placing the fish into a novel tank. Even the pouring method is not devoid of aversive features: lowering the water in the home tank and pouring the fish from that tank can provide plenty of potentially aversive cues. A true solution for human-handling-related issues is to not have human handling at all. This is what home tank monitoring may accomplish, a topic I consider below.

Monitoring and testing the behaviour of experimental animals without human interference has been considered for rodents. There have been a variety of home-cage monitoring systems developed, some of which were/are available commercially, e.g., the Intellicage [91], or are proprietary, e.g., the SmartCube (https://www.psychogenics.com/in-vivo-ai-platforms/smartcube, accessed on 25 February 2026; see also [92]). The literature on this topic has expanded, as scientists using mice and rats started to recognize the need to minimize human-handling-induced problems (for a recent review, see [93]). However, home-tank monitoring systems have not been commercially available for the *zebrafish*, and the literature on this topic for *zebrafish* is rather sparse as well (but see [94,95]). Perhaps one reason for this is that the analysis of learning in the home tank of *zebrafish* is not easy to achieve. The currently available commercial high-density rack systems often employ tiny tanks with odd shapes and little room around the tank for any additional equipment that may be required for learning studies. For example, movement detection devices, including cameras or photo-beams, would be difficult to employ on these racks simply due to a lack of room for the required equipment. Devices that would deliver visual cues as CS and rewards (US), including food, may also be difficult to attach to tanks of commercially available high-density rack systems. To circumvent these problems, we designed a stand-alone apparatus, a 40-litre glass tank that was equipped with LEDs to provide CSs and food delivery magazines to provide the US [21]. The system was designed so that the experimental subject could live in the apparatus for several days. We called it a semi-automated system, as the experimental subject did have to be transported to the tank manually. Nevertheless, after having been placed in this tank, the experimental fish was left alone, and all experimental procedures, training and behaviour monitoring included, were conducted with no human interference. Using this semi-automated learning apparatus, we were able to demonstrate the acquisition of the CS–US association as well as colour discrimination memory in *zebrafish* [21]. A potential drawback of this semi-automated method was that each experimental fish had to be monitored separately; i.e., the fish were isolated for the period of the experiment (5 days, in this case). Isolation may, itself, induce anxiety, as *zebrafish* is a highly social shoaling fish that prefers staying in close proximity to its conspecifics [63,64,96,97].

At this point, I can only theorize how one could improve home-tank monitoring systems to circumvent the issues mentioned above, the last topic of this review I briefly consider. As mentioned before, one issue is the physical space, size, and shape of the test tank, and the room around it to accommodate the attachment of equipment. The other is the fact that *zebrafish* is a social species, but most learning studies require training and monitoring the responses of *zebrafish* alone, i.e., one fish at a time. I deal with the former issue first, which is easier to solve than the latter one. Could we, or should we, use larger tanks in our high-density *zebrafish* system racks? The answer to both of these questions is yes. Although the “industry-standard” is still small tanks, systematic studies exploring the questions of what may be the optimal tank size and/or optimal fish density have started to provide evidence that crowding *zebrafish* into tiny tanks is not a good idea [98]. Increasing the tank size and decreasing the fish density may reduce the physiological and psychological stress for *zebrafish*, and thus may make them exhibit reduced fear and anxiety responses [98]. In our facility, we use 50-litre glass tanks that we specifically developed to fit on our Aquaneering *zebrafish* racks. The tanks not only fit physically, but they also “plug into” the filtration system of the rack. That is, they have pipes whose water intake location is near the bottom of the tank allowing debris to exit (autoflush) through the pipe. The pipe then lifts the water and releases it near the water surface into the collecting tray of the rack system positioned behind the tank. From there, the old/dirty water goes through the regular filtration steps of the rack system (physical, biological, and chemical filtration followed by UV-light sterilization). After these filtration steps, the clean water enters the large glass tank from the top through small silicone tubes. The size of this tank and the space around it would make it optimal for home tank learning studies. The second problem, i.e., the question of solitary versus group housing for the fish, is more difficult to address. To acquire a strong-enough CS–US association memory, *zebrafish* are often required to receive at least 10 training trials (for review and examples, see [14,16,18,19]). To ensure each fish is trained properly, the fish are trained individually, i.e., one at a time. Furthermore, to follow the acquisition of CS–US association memory, investigators often monitor the behavioural responses of their fish, which, again, requires testing one fish at a time. This requires housing the fish singly, which is potentially fear/anxiety-inducing [96,97]. Nevertheless, one could argue that physical isolation does not necessarily mean social isolation, as fish housed next to each other may see one another, and singly housed fish in a recirculating tank system (such as most commercial high-density *zebrafish* rack systems) will also be able to perceive chemical (olfactory) cues of each other. However, physical isolation may still have negative consequences. Could we train and test group-housed fish, that is, fish that is housed in their home tank in the usual manner? There are multiple issues we need to address to be able to answer yes to this question. But, first, let me note that group training and group testing for learning and memory performance have been attempted with laboratory organisms (see, e.g., [99]). The problem with this approach is that one cannot dissociate CS–US associative learning from social learning. In other words, some fish may appear to perform well in the learning test not because they actually acquired CS–US associative memory, but because they learned to follow a leader to obtain the desired US.

Having considered this issue, let me focus on a more basic question: can we individually distinguish *zebrafish*? This is a crucial and fundamental requirement in a learning study if we want to train fish and follow the acquisition of memory for each of them separately. Although *zebrafish* look quite alike, i.e., variations in individual physical features are not easy to observe/quantify/detect, fish can be artificially marked. We developed a colour dye injection-based marking method (using non-toxic food colouring dyes) [100]. But other methods, including the injection of small and flexible elastomers or tags, have also been successfully employed [101]. Computerized video-tracking systems are already commercially available that can detect multiple colours and could thus distinguish, and track the swim path of, multiple fish at a time (for a review, see [13]; for a comparison of video-tracking and other behavioural quantification methods for *zebrafish*, see also [102]). Whether the use of such systems is practically feasible for our purposes has to be empirically established. Nevertheless, monitoring the activity of individual fish in a group housing environment should be possible in principle. The second problem is more difficult: how could we avoid mixing up CS–US acquisition and social learning? One solution would be to create conditions that would allow us to train *zebrafish* individually in the home tank, even though we group-house them. This may be accomplished by designing and implementing a tunnel system that would allow the group-housed fish to move spontaneously from one compartment, the group-housing side, to the test/training side of the tank. This tunnel could be equipped with a sensor that would detect the passing of a fish through the tunnel, and, once one fish is deeper inside, an automated door could lower and prevent the single fish from turning back. Once the fish is in the test/training side, training would commence in the usual manner (CS–US co-presentation). Subsequently, the door could open and close again once the trained fish has moved back to its home compartment. Although, in principle, the above may sound easy, in practice, one would have to establish a design that would allow the experimenter to minimize the possibility of multiple fish entering the tunnel and/or un-trained fish entering the tunnel when the test fish is returning to the home compartment. Clearly, there will be several practical hurdles one would have to go through before such a home-tank system would work. But, again, in principle, I believe such systems are possible and will be designed in the future. These systems then would completely solve the issues associated with human handling.

## 9. Conclusions

Although the *zebrafish* is decades behind the most popular biomedical research organism, the house mouse, in terms of our knowledge of its cognitive abilities, and also in terms of our methods with which we can test these abilities, research in this area is rapidly evolving. Our own efforts on laying the foundation of how to conduct learning studies with *zebrafish*, that is, our focus on finding out how to motivate these fish in learning tasks, have already paid dividends. We now know that the type of food and the method of its delivery can make a difference in terms of how rewarding this unconditioned stimulus is in learning studies with the *zebrafish*. We now also know that limiting food intake prior to associative training trials with food as a US is counterproductive in the case of *zebrafish*. Furthermore, we, and others, have discovered appetitive stimuli other than food, which have now been successfully utilized as a US in learning studies with *zebrafish*. And we have learned a lot about how to reduce the effects of confounding factors, including fear and anxiety, in appetitive conditioning tasks with *zebrafish*. For several of these developments and discoveries, an understanding of the ethology and ecology of the *zebrafish* played a role. Indeed, others, and I too, have argued that learning about the habitat and natural behaviour of our study species, including the *zebrafish*, and borrowing from nature will continue to help us in this context. I believe the rapid progress in the analysis of learning and memory of *zebrafish* will continue, and the *zebrafish*, given its simplicity coupled with its numerous evolutionarily conserved features, will become one of the most frequently employed research species in the preclinical and biomedical, as well as basic research of cognition studies alike.

## Data Availability

No new data were created or analyzed in this study. Data sharing is not applicable to this article.

## Abbreviations

The following abbreviations are used in this manuscript:

US	Unconditioned Stimulus
CS	Conditioned Stimulus
2D	Two-dimension
DOPAC	3,4-Dihydroxyphenylacetic acid
